# Identification of antibiotic‐resistant pathogens and virulence genes in *Escherichia coli* isolates from food samples in the Dhaka University campus of Bangladesh

**DOI:** 10.1002/fsn3.3896

**Published:** 2023-12-27

**Authors:** Progati Bakshi, Anindita Bhowmik, Sunjukta Ahsan, Sharmin Rumi Alim

**Affiliations:** ^1^ Institute of Nutrition and Food Science University of Dhaka Dhaka Bangladesh; ^2^ Department of Food Engineering Bangabandhu Sheikh Mujibur Rahman Science and Technology University Gopalganj Bangladesh; ^3^ Department of Microbiology University of Dhaka Dhaka Bangladesh

**Keywords:** antibiotic resistance, Bangladesh, chicken curry, Dhaka University, *Escherichia coli*, foodborne illness, pathogenic bacteria, potato smash, virulence genes

## Abstract

The presence of antibiotic‐resistant pathogens in food is a serious public health concern nowadays. This study focuses on the isolation and characterization of potentially pathogenic *Escherichia coli* and antimicrobial‐resistant pathogens in chicken curry and potato smash samples collected from the canteens and cafeteria of Dhaka University in Bangladesh. Isolates were identified by their cultural, morphological, and biochemical tests (motility indole urease test, Kliger's iron agar test, catalase test, oxidase test, methyl red and Voges‐Proskauer tests). The antibiotic susceptibility test was done by the disk diffusion method. The range of total bacterial count in the potato smash and chicken curry samples was from 1.4 × 10^4^ to 1.6 × 10^8^ CFU/g and from 2.4 × 10^3^ to 2.6 × 10^6^ CFU/g, respectively. *Escherichia coli*, *Salmonella*, *Vibrio*, *Klebsiella*, *Citrobacter*, *Enterobacter*, *Proteus*, *Clostridium*, *Staphylococcus*, *Streptococcus*, *Micrococcus*, *Bacillus*, and *Sarcina* strains were isolated in both samples. Isolates were highly resistant to ampicillin (90.90%) followed by colistin (52.27%), azithromycin (27.27%), and tetracycline 25%. *Proteus* species had the highest rate of multiple antibiotic resistance (MAR; 62.5%), followed by *Citrobacter* species (50%). The isolated *E. coli* strains were further analyzed through PCR assay to detect virulent genes (EPEC: *eaeA* 229 bp, *bfpA* 450 bp, ETEC *elt* 322 bp, EHEC *hylA* 534 bp, and EIEC *ial* 320 bp). One *E. coli* isolate had the *eae*A target gene under EPEC pathotypes. *Escherichia coli*, as a fecal indicator, may indicate fecal contamination or poor and unhygienic food handling. The findings recommend further investigations to identify potential mechanisms of contamination and preventive measures to improve the food safety level in the canteens and restaurants.

## Graphical abstract

*Escherichia coli*, *Salmonella*, *Vibrio*, *Klebsiella*, *Citrobacter*, *Enterobacter*, *Proteus*, *Clostridium*, *Staphylococcus*, *Streptococcus*, *Micrococcus*, *Bacillus*, and *Sarcina* were isolated from the chicken curry and potato smash samples. The most resistant antibiotic was ampicillin (90.90%), followed by colistin (52.27%), azithromycin (27.27%), and tetracycline (25%). *Proteus* species had the highest rate of multiple antibiotic resistance (62.5%), followed by *Citrobacter* (50%). The presence of the *eaeA* target gene under EPEC pathotypes in isolated *E. coli* strains may indicate insufficient and unclean food handling.

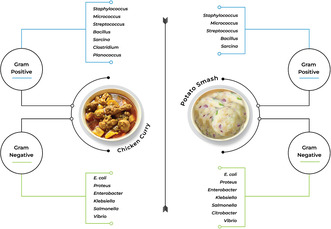

## 1 INTRODUCTION

Foodborne diseases cover a wide spectrum of illnesses and become an increasing public health concern. Every year, pathogen‐contaminated food causes around 600 million instances of food‐related illness and 420,000 fatalities worldwide (WHO, 2022). However, the emergence of antibiotic‐resistant pathogens in food poses further threats to food safety and public health (Caniça et al., 2019; Pérez‐Rodríguez & Mercanoglu Taban, 2019).

Enteric pathogens, notably *Escherichia coli* O157:H7, *Campylobacter*, and *Salmonella* are primarily spread by food (Erickson & Doyle, 2007; Martinez et al., 2007). A number of *E. coli* clones have virulence properties and can cause a wide range of diseases. The six major classes of intestinal *E. coli* are enteropathogenic *E. coli* (EPEC), enterohemorrhagic *E. coli* (EHEC), enterotoxigenic *E. coli* (ETEC), enteroaggregative *E. coli* (EAEC), enteroinvasive *E. coli* (EIEC), and diffusely adherent *E. coli* (DAEC) (Nataro & Kaper, 1998). *Escherichia coli* is the most frequent cause of water‐ and foodborne diarrhea, which led to infant deaths in low‐ and lower middle‐income nations (Turner et al., 2006). For instance, it was stated that 30 million individuals in Bangladesh were afflicted by *E. coli*‐related foodborne diseases (Rahman et al., 2018). Focusing on the microbiological aspect of food, the presence of *E. coli* may indicate poor and unhygienic food handling and fecal contamination. Therefore, the detection of *E. coli* and its virulence properties is an important consideration while assessing the microbiological quality of food (Hossain et al., 2023; Sherikar & Bachhi, 2011). Furthermore, the presence of pathogens, including *E. coli*, in food could be attributable to cross‐contamination, temperature abuse, and unhygienic food handling, which are important aspects of food safety (Todd et al., 2010).

Dhaka, the capital of Bangladesh, is one of the world's most densely populated cities, with a population of over 10 million people (BBS, 2022). The University of Dhaka is one of the busiest areas in Dhaka city. Food in this area is comparatively less expensive and consumed not only by resident students/faculties, but also by nonresident students/faculties and people from different sociodemographic backgrounds. Moreover, in line with the positive sociodemographic change in Bangladesh, there has been an increasing trend of ready‐to‐cook food consumption among people in Dhaka city. For example, there has been a spring up in the number of restaurants employing more than two million people in this sector in the last decade (Byron & Jahid, 2021). Therefore, contamination of foods even from a single source/canteen could potentially affect a large number of consumers and pose a potential gradient for a public health crisis.

The occurrence of multidrug‐resistant bacteria in food is becoming more common in Bangladesh. Previous research found multiple drug‐resistant (MDR) *Salmonella* spp. *Vibrio* spp. in poultry, which is a vital protein source for Bangladeshis (Akond et al., 2008, 2012; Barua et al., 2012; Parvej et al., 2016). This could lead to poor efficacy of the commonly used antibiotics and subsequently public health crisis (Hoque et al., 2020). Intake of antibiotics without the prescription of registered physicians and not maintaining the schedule is mainly attributed to growing antibiotic resistance (Chowdhury et al., 2019; Hoque et al., 2015). However, antibiotic resistance due to the consumption of foods containing multidrug‐resistant pathogens is often overlooked.

Several studies in Bangladesh examined the microbiological quality of various food items sold at different university campuses (Biva et al., 2019; Khan et al., 2015; Younus et al., 2020). However, most of the studies focused on bacterial load and characterization, whereas research on antibiotic resistance and the presence of virulence genes was scarce. Therefore, the purpose of this study is to investigate the existence of pathogenic bacteria, their antibiotic resistance traits, and the presence of virulence genes in *E. coli* isolates in commonly consumed foods, like potato smash and chicken curry, from the canteens at the Dhaka University. Findings could be useful to update existing evidence and implement food safety policies to prevent and control foodborne illness.

## 2 MATERIALS AND METHODS

### 2.1 Sample collection

Samples included two food items: potato smash and chicken curry. Potato smash is a traditional food item, whereas chicken is one of the most consumed protein source in Bangladesh. A total of 50 samples (25 samples of potato smash and 25 samples of chicken curry) were collected from 5 different food delivery points. These lunch dishes were typically prepared at noon around 12 o'clock. In order to prevent cross‐contamination from exposure to the environment or longer storage times after cooking, samples were taken as soon as they were prepared. The time needed for preparation, plating, incubation, and isolation for each sample was balanced by collecting samples twice a week. Samples were collected using sterile containers covered with aluminum foil paper.

### 2.2 Preparation of samples

The samples were taken out from the sterile containers and placed in a sterile Petri dish. Ten grams of each sample was mixed with previously prepared 90 mL peptone water and plugged with cotton, and the flask was shaken for homogenizing the samples. In accordance with the recommendations of the American Public Health Association (APHA), the sample homogenate was diluted at 10‐fold dilution up to 10^−4^ (Greenberg, 1992).

### 2.3 Bacteriological studies

The spread plate method was used to isolate the bacterium. Bergey's Manual of Determinative Bacteriology (9th Edition) was followed to study cultural, morphological, and biochemical traits in order to identify the isolated bacteria (Bergey, 1994).

Different types of nonselective and selective agar were used for isolation; for example, plate count agar (PCA) for the viable count, MacConkey (MC) for gram‐negative enteric bacteria, *Salmonella–Shigella* (SS) for various species of *Salmonella* and *Shigella*, eosin–methylene blue (EMB) for coliform bacteria, and thiosulfate–citrate–bile–sucrose (TCBS) for *Vibrio*. For the detection of *Clostridium* and *Listeria*, cooked meat media was employed, and fungal development was monitored using potato dextrose agar (PDA). Bacterial colonies grown at 37°C on different types of media were collected and maintained in nutrient‐slant agar for further analysis. They were studied for their color, shape, size, margin, elevation, and surface (Tables S2 and S3). Morphological characteristics were examined by gram staining and with the help of light microscopy by an oil immersion microscope. Biochemical tests such as Kliger's iron agar (KIA) test, motility indole urease (MIU) test, catalase test, oxidase test, methyl red, and Voges–Proskauer test were performed for the identification of bacterial isolates.

### 2.4 Antibiotic sensitivity test

The antibiotic sensitivity test was done by the disk diffusion method. Mueller–Hinton agar (MHA) and Mueller–Hinton broth (MHB) were used as media. Colonies of selected isolates were collected by loop from fresh subcultured media and inoculated into 5 mL MHB, mixed well by vortex mixture. The optical density of the inoculated broth was measured in the spectrophotometer. The turbidity of the saline solution was compared with standard MacFarlane 0.5 solution. A cotton swab was dipped into the turbid MHB and a lawn was prepared on MHA. Specific antibiotic disks were placed on the inoculated MHA media using sterile forceps and disks were gently pressed onto the agar surface. After 18–20 h of incubation, zones of inhibition were observed on inoculated MHA. A clear zone indicates susceptibility of bacteria to the specific antibiotic and no clear zone indicates resistance to the antibiotic. The Clinical and Laboratory Standard Institute's (CLSI) guideline was used to interpret the inhibition zones and classify the isolates as resistant (<7 mm), intermediate (7 mm), or sensitive (>7 mm) (Hsueh et al., 2010). Ampicillin (AMP 25 μg), azithromycin (AZM 30 μg), ciprofloxacin (CIP 5 μg), colistin (CL 10 μg), chloramphenicol (C 30 μg), gentamicin (GEN 10 μg), levofloxacin (LE 5 μg), and tetracycline (TE 30 μg)‐ total eight commercially available antibiotic disks were used against each isolate. The multiple antibiotic resistance (MAR) index was calculated as the ratio of the number of antibiotics to which the isolate showed resistance to the total number of antibiotics to which the isolate was exposed (Krumperman, 1983).

### 2.5 Detection of the virulent gene by PCR

A single colony from pure *E. coli* culture was collected using sterile toothpicks and mixed into 50 μL sterile deionized water. The further procedure included heat shock for 5 min at 95°C in a PCR block (Table S12), centrifugation (10,000 *g*) of the heat‐lysed cells for 3 min, and collection of supernatants to be used as a DNA template. Amplification of the desired gene in the selected *E. coli* isolates was carried out by PCR (Hegde et al., 2012). An array of five primers (Table 1) of the following target genes—*eaeA* 229 bp, *bfpA* 450 bp, *elt* 322 bp, *ial* 320 bp, and *hlyA* 534 bp—was used to detect respective four different categories (EPEC, ETEC, EIEC, and EHEC) of *E. coli*.

**TABLE 1:** Primer sequences for the identification of *Escherichia coli* pathotypes.

Reference strain	Primer sequence	Target gene	*T*ᵐ (°C)	Amplicon size (bp)	Reference
EPEC	5′–TGATAAGCTGCAGTCGAATCC–3′	*eaeA*	54.8	229	Hegde et al. (2012)
	5′–CTGAACCAGATCGTAACGGC–3′		55.7		
	5′–CACCGTTACCGCAGGTGTGA–3′	*bfpA*	59.9	450	
	5′–GTTGCCGCTTCAGCAGGAGT–3′		60.6		
ETEC	5′–CTCTATGTGCACACGGAGC–3′	*elt*	53.3	322	
	5′–CCATACTGATTGCCGCAAT–3′		55.8		
EIEC	5′–CTGGTAGGTATGGTGAGG–3′	*ial*	51.2	320	
	5′–CCAGGCCAACAATTATTTCC–3′		51.9		
EHEC	5′–GCATCATCAAGCGTACGTTCC–3′	*hlyA*	56.5	534	
	5′–AATGAGCCAAGCTGGTTAAAGCT–3′		57.5		

The PCR was performed using different conditions, including initial denaturation at 95°C for 10 min, denaturation at 95°C for 1 min, annealing at 55°C for 1 min, and extension at 68°C for 1 min. These three steps were repeated sequentially for 35 cycles with a final extension at 68°C for 10 min.

## 3 RESULTS

### 3.1 Morphological, phenotypic, and biochemical traits

The bacterial count in the potato smash samples extended from 1.4 × 10^4^ to 1.6 × 10^8^ CFU/g. Among five food delivery points, total bacterial loads in the samples from four were unsatisfactory, whereas the other one lies near the edge of the acceptable range. For the chicken curry samples, the bacterial count was from 2.4 × 10^3^ to 2.6 × 10^6^ CFU/g. Considering both food items, four service delivery points represented unsatisfactory bacterial count, whereas only one had a satisfactory count (Table 2).

As the presence of any enteric bacteria in food is hazardous, the growth of these bacteria in any selective media indicates unsatisfactory results. Among the samples, the maximum growth on the MacConkey medium was found for potato smash (8.0 × 10^5^ CFU/g), whereas for chicken curry samples, the intensity of growth extended from moderate (1.14 × 10^3^ CFU/g) to no count. On the EMB media, a distinctive metallic green sheen that pointed to the presence of *E. coli* bacteria was visible. All samples exhibited positive growth in cooked meat and potato dextrose agar (Table S1).

Among the 62 discrete isolates (36 from potato smash and the rest 26 from chicken curry samples), 43 of the total isolates were gram‐negative rod shaped, 14 were gram‐positive coccus, and only 5 were gram‐positive rods (Table S4 and S5). Different biochemical tests were conducted to identify bacterial species in the samples (Table S6 and S7).

**TABLE 3:** Multiple antibiotic resistance (MAR) index of isolated bacteria.

Bacteria spp.	MAR%
*Escherichia coli*	37.50
*Enterobacter* spp.	37.50
*Salmonella* spp.	37.50
*Proteus* spp.	62.50
*Vibrio* spp.	25.00
*Klebsiella* spp.	12.50
*Citrobacter* spp.	50.00
*Bacillus* spp.	25.00
*Micrococcus* spp.	37.50
*Staphylococcus* spp.	37.50
*Streptococcus* spp.	50.00
*Planococcus* spp.	37.50
*Sarcina* spp.	37.50

### 3.2 Identification of the isolates

**FIGURE 1:**
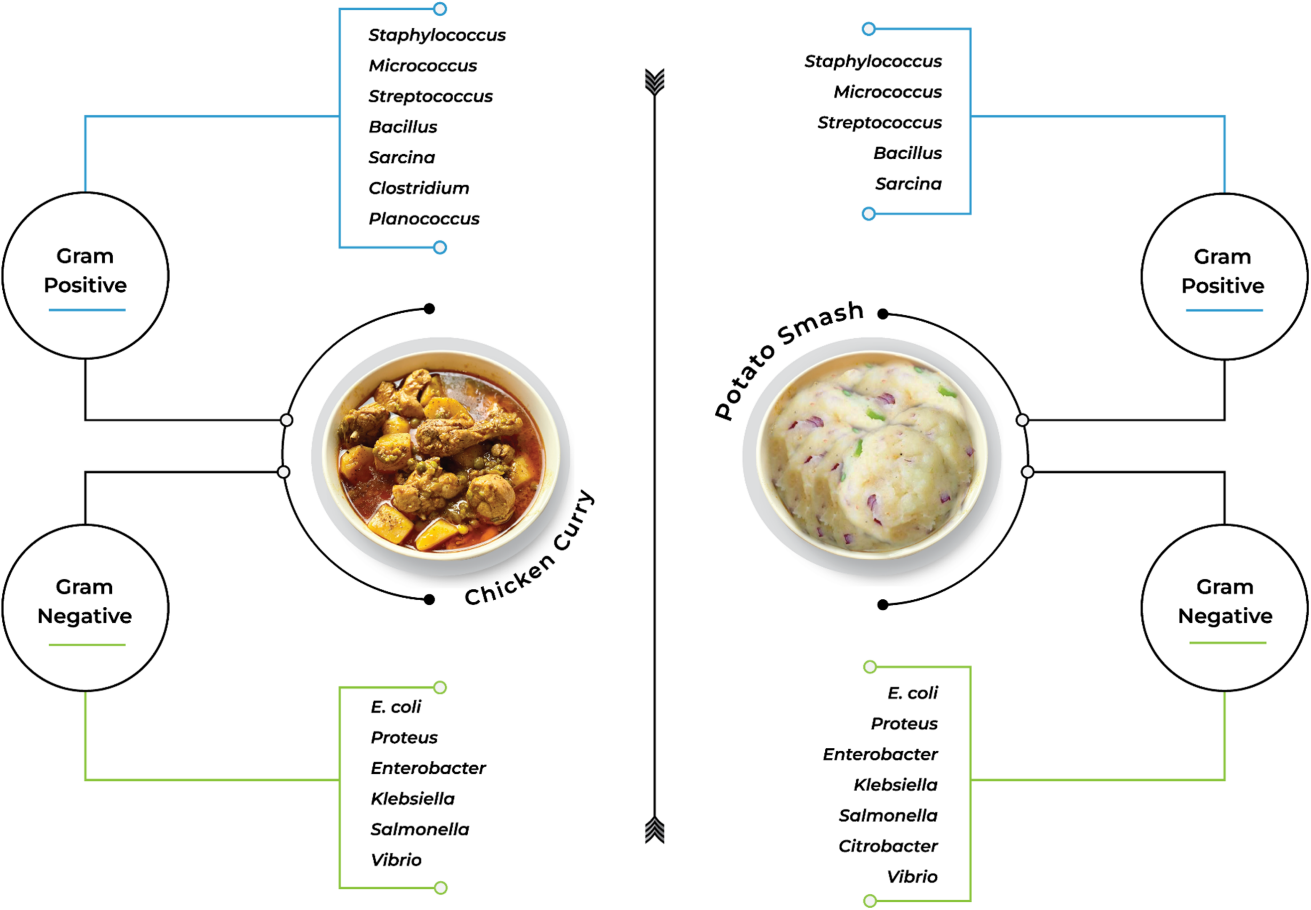
Bacterial isolates identified in the chicken curry and potato smash samples.

This study revealed the presence of various gram‐negative bacteria namely *E. coli* (*n* = 14), *Salmonella* (*n* = 7), *Vibrio* (*n* = 3), *Citrobacter* (*n* = 1), *Klebsiella* (*n* = 6), *Enterobacter* (*n* = 5), and *Proteus* (*n* = 7). Among gram‐positive strains, *Staphylococcus* (*n* = 4), *Streptococcus* (*n* = 4), *Micrococcus* (*n* = 3), *Bacillus* (*n* = 4), *Planococcus* (*n* = 1), *Clostridium* (*n* = 1), and *Sarcina* (*n* = 2) were found (Figure 1, Table S8 and S9).

### 3.3 Antibiotic susceptibility and MAR indices

For the analysis of the antibiotic susceptibility patterns, 26 gram‐negative and 18 gram‐positive isolates were selected. All the gram‐negative isolates were resistant to at least one antibiotic, and 18.75% were resistant to more than three antibiotics. On the other hand, almost half of the gram‐positive isolates were resistant to at least two antibiotics, and only two isolates represented sensitivity against all eight antibiotics (Tables S10 and S11).

**FIGURE 2:**
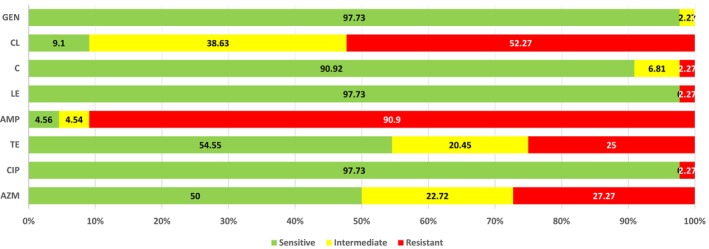
Resistance rates to tested antibiotics.

The findings showed that all of the tested isolates are highly resistant to ampicillin (90.90%) followed by colistin (52.27%), azithromycin (27.27%), and tetracycline 25%. While ciprofloxacin (2.27%), levofloxacin (2.27%), chloramphenicol (2.27%), and gentamicin (0.00%) showed less resistance (Figure 2).

The *Proteus* spp. showed the highest MAR percentages (62.5%) followed by *Citrobacter* spp. (MAR 50%). Most of the strains showed multiple resistance against azithromycin, tetracycline, ampicillin, and colistin (Table 3).

**TABLE 2:** Total count of viable bacteria in the potato smash and chicken curry samples.

Sample	Service delivery points	PCA (CFU/g)	MacConkey (CFU/g)	EMB (CFU/g)
Potato smash	A	2.8 × 10^5^ Unsatisfactory	2.0 × 10^3^ Moderate	1.0 × 10^5^ High
	B	1.6 × 10^8^ Unsatisfactory	1.1 × 10^4^ High	2.6 × 10^3^ Moderate
	C	1.4 × 10^4^ Acceptable	1.4 × 10^4^ High	2.6 × 10^4^ High
	D	1.2 × 10^7^ Unsatisfactory	4.8 × 10^3^ Moderate	8.0 × 10^3^ Moderate
	E	2.0 × 10^6^ Unsatisfactory	8.0 × 10^5^ High	8.0 × 10^5^ High
Chicken curry	A	2.0 × 10^6^ Unsatisfactory	1.02 × 10^3^ Moderate	3.0 × 10^3^ Moderate
	B	2.6 × 10^6^ Unsatisfactory	NIL No growth	4.0 × 10^2^ Low
	C	2.4 × 10^3^ Good	NIL No growth	2.0 × 10^3^ Moderate
	D	2.0 × 10^6^ Unsatisfactory	1.14 × 10^3^ Moderate	1.6 × 10^3^ Moderate
	E	1.2 × 10^5^ Unsatisfactory	NIL No growth	NIL No growth

*Note*: Remark: According to the International Commission on Microbiological Specifications for Foods. <10^4^ = Good, <10^5^ = Acceptable, ≥10^5^ = Unsatisfactory. Intensity of growth:  < 10^3^ = low growth, <10^4^ = moderate growth, >10^4^ = high growth (The average count of five duplicates of the same samples has been taken).

### 3.4 Detection of pathotypes among *E. coli* isolates

**FIGURE 3:**
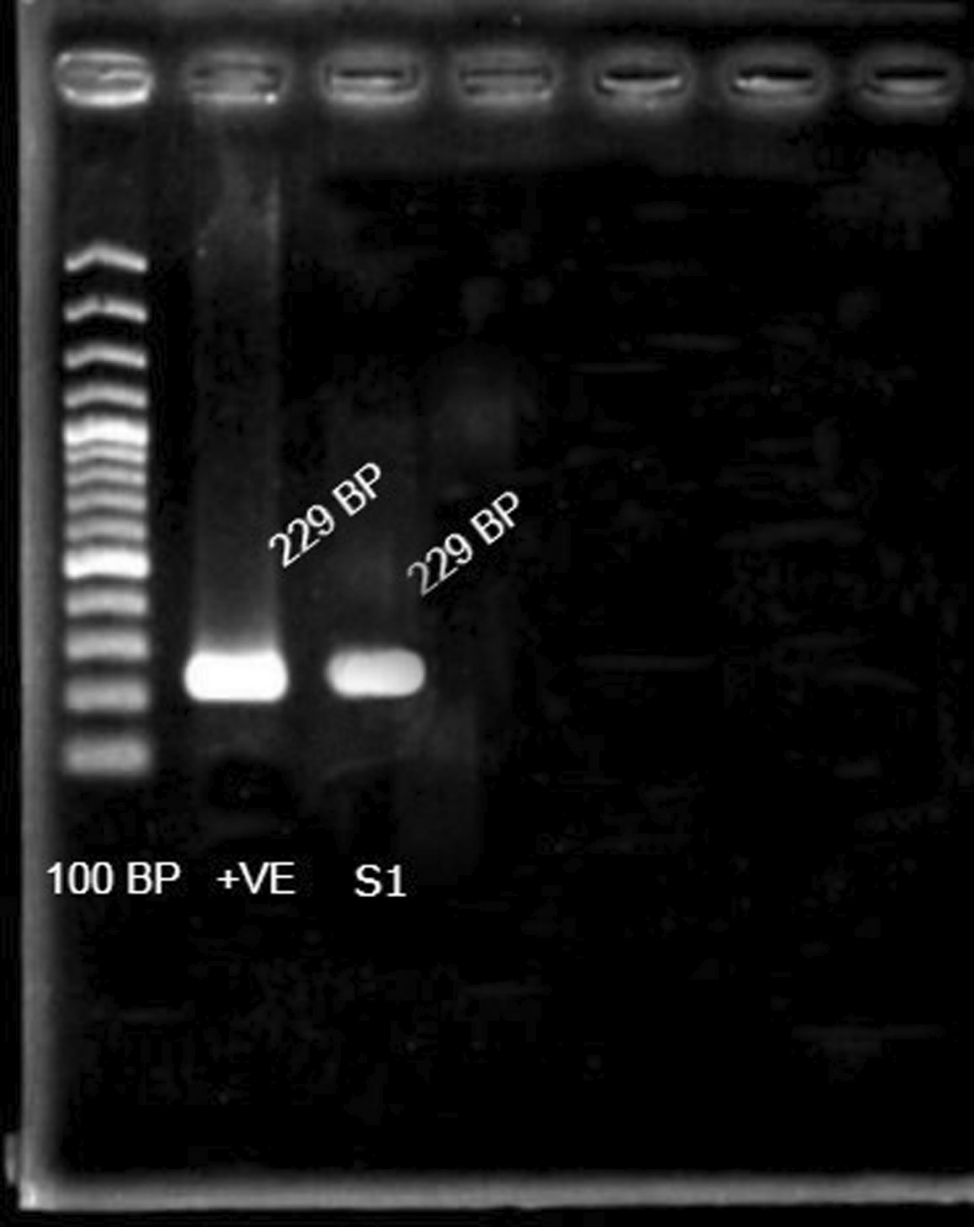
Detection of *eaeA* gene by agarose gel electrophoresis. Lane 1, 100‐bp DNA ladders; Lane 2, *eaeA* virulent gene (229 bp) used as positive control; Lane 3, PCR result of the S1 coded E. coli isolate. Our presumptive strain created the same product size (229 bp) and band intensity as the *eaeA* primer (Lane:2) which confirmed the presence of *eaeA* virulent gene in the tested E. coli isolate. (Lane: 3)

The sample denoted as S1 (BMCPG1) showed a positive isolate harboring the *eaeA* (229 bp) gene under EPEC pathotypes (Figure 3).

## 4 DISCUSSION

In this study, we assessed the microbiological quality of chicken curry and potato smash samples available at the Dhaka University campus. A wide variety of bacteria was identified. Among the gram‐negative isolates, *E. coli*, *Salmonella*, *Vibrio*, *Citrobacter*, *Klebsiella*, *Enterobacter*, and *Proteus*, and the following gram‐positive isolates—*Staphylococcus*, *Streptococcus*, *Micrococcus*, *Bacillus*, *Clostridium*, and *Sarcina* were found. According to our findings, chicken curry contained less viable bacteria than potato smash. A possible reason could be the cooking method of chicken curry which requires a long time of heating under high temperature. A recent study discovered that instant‐cooked food had fewer microbes than raw or stored food (Heetun et al., 2015; Hosen & Afrose, 2019).

The traditional method for preparing common potato smash requires raw onion, green chili, and coriander leaf which could be a possible source of contamination if proper hygiene is not maintained. For instance, the predominant number of *E. coli* and *Enterobacter* in the food samples could be due to the usage of raw vegetables or raw components.

Meat contamination by spore‐forming bacteria could occur during slaughtering, processing, and storage, primarily due to poor sanitary and handling practices. The presence of *Clostridium* spp. in chicken curry indicates unhygienic or lack of proper processing of raw meats. A diffusive yellow *Vibrio* colony on TCBS agar was found in the potato smash sample. This could happen as a result of cross‐contamination from other sources, such as raw materials, tainted water, utensils, or improper handling (Malcolm et al., 2018; Nawas et al., 2012).

The virulence genes in *E. coli* were detected to identify the natural differences in pathogenicity between isolates of the same species. In our study, we tested 15 isolates against the following reference strains and target genes: EPEC (*eaeA* 229 bp, *bfpA* 450 bp), ETEC (*elt* 322 bp), EIEC (*ial* 320 bp), and EHEC (*hlyA* 534 bp). Among them, one of the tested *E. coli* isolates (from the potato smash sample from canteen A) showed the presence of the *eaeA* (229 bp) gene under EPEC pathotypes. These EPEC pathotypes of *E. coli* may cause cholecystitis, bacteremia, cholangitis, urinary tract infection (UTI), traveler's diarrhea, and other clinical illnesses like as neonatal meningitis and pneumonia (Geurtsen et al., 2022).

In recent decades, evidence of antibiotic‐resistant properties among foodborne pathogens is increasing (Akbar & Anal, 2014a; Van et al., 2007). The MAR index is a useful approximation for determining if isolates are from a high or low antibiotic use zone (Davis & Brown, 2016). According to our findings, the MAR percentage of *E. coli* was determined to be 37.5%, which incorporates an extensive antibacterial drug resistance. Ampicillin, colistin, azithromycin, and tetracycline demonstrated high resistance in all of the isolates. A similar study was carried out to look into the antimicrobial sensitivity profile of *E. coli* and its pathogenic strain O157 to common antibiotics and their presence in poultry where *E. coli* isolate showed resistance to ampicillin, tetracycline, gentamicin, and chloramphenicol (Akbar et al., 2014). We also discovered that the third‐generation antibiotic ciprofloxacin is ineffective against *Proteus* species and *Salmonella* species. Levofloxacin and gentamicin are still among the most effective and safest antibiotics as listed by the World Health Organization. We also did not find any resistance against them.

In our study, isolates of *Proteus*, *E. coli*, *Enterobacter*, *Staphylococcus*, and *Bacillus* were found to be completely resistant to ampicillin and colistin. A study on dairy beverages available in the Dhaka University campus represented similar findings, where *E. coli*, *Pseudomonas*, *Klebsiella*, *Proteus*, *Shigella*, *Aeromonas*, *Micrococcus*, *Bacillus*, and *Clostridium* were found resistant against both ampicillin and colistin (Biva et al., 2019).

Considering high bacterial counts and the presence of multidrug‐resistant pathogens, the quality of the food samples could be considered unacceptable. Identified pathogens showed resistance against both conventional and new‐generation antibiotics (except levofloxacin, ciprofloxacin, and gentamicin). The emergence of multidrug‐resistant bacteria is a serious public health threat; therefore, preventive measures are required to improve the situation. Foodborne infections are mostly related to unsanitary procedures and the use of infected devices and materials in food processing (Akbar & Anal, 2014b; Ruban et al., 2012). Hence, food should be prepared with the least possible manual contact, clean utensils, and clean surface contact to prevent cross‐contamination. Every canteen should be routinely monitored using the Hazard Analysis Critical Control Point (HACCP) process to prevent foodborne diseases. Moreover, improved investigation, correct diagnosis, reporting to public health authorities, training, and interventions should be prioritized at the state and local levels.

## 5 CONCLUSION

According to our findings, a high microbial load was found in the samples of chicken curry and potato smash collected from different canteens at the Dhaka University. Given the very elevated bacterial counts and the prevalence of multidrug‐resistant foodborne pathogens, the microbiological quality and safety of the items were subpar. The presence of the virulence *E. coli* genes indicated fecal contamination which was quite unacceptable and requires further attention. Therefore, further investigations are recommended to examine more food items, identify mechanisms of contamination, and implement preventive measures.

## AUTHOR CONTRIBUTIONS

**Progati Bakshi:** Data curation (lead); formal analysis (equal); funding acquisition (lead); investigation (equal); methodology (equal); project administration (equal); resources (equal); software (lead); writing – original draft (lead); writing – review and editing (equal). **Anindita Bhowmik:** Formal analysis (supporting); methodology (supporting); software (supporting); writing – review and editing (supporting). **Sunjukta Ahsan:** Formal analysis (supporting); investigation (supporting); methodology (supporting); resources (equal); supervision (supporting); writing – review and editing (equal). **Sharmin Rumi Alim:** Conceptualization (lead); formal analysis (equal); funding acquisition (supporting); investigation (equal); methodology (equal); project administration (equal); resources (equal); supervision (lead); validation (lead); visualization (lead); writing – review and editing (equal).

## CONFLICT OF INTEREST STATEMENT

The authors declare no conflicts of interest.

## ETHICS STATEMENT

Not applicable.

## Supporting information


Table S1.

## Data Availability

Supplementary data have been provided.
