# Supplementary material for: Identification of antibiotic‐resistant pathogens and virulence genes in Escherichia coli isolates from food samples in the Dhaka University campus of Bangladesh
Source: Food Sci Nutr. 2023 Dec 27;12(3):1995–2002. doi: 10.1002/fsn3.3896 (PMC10916665; doi:10.1002/fsn3.3896)
Supplement: Supplementary file 1 — Table S1. [file FSN3-12-1995-s001.docx]

| Supplementary Table 1: Results of the extent of bacterial growth using TCBS, Cooked meat media, SS agar, and PDA | | | | |
| --- | --- | --- | --- | --- |
| Service Delivery Points | Growth in TCBS* | Turbidity and gas formation in cooked meat media | Growth in SS* | Fungal growth on PDA |
| Sample: Potato Smash | | | | |
| A | Nil | +++ | ++ | +++ |
| B | Nil | ++ | Nil | +++ |
| C | Nil | +++ | +++ | +++ |
| D | Nil | +++ | +++ | ++ |
| E | ++ | +++ | +++ | +++ |
| Sample: Chicken Curry | | | | |
| A | Nil | +++ | ++ | +++ |
| B | Nil | + | Nil | + |
| C | Nil | +++ | Nil | ++ |
| D | Nil | +++ | ++ | +++ |
| E | Nil | + | Nil | + |

| Supplementary Table 2: Colony characteristics of the isolates from Potato Smash | | | | | | |
| --- | --- | --- | --- | --- | --- | --- |
| **Isolates** | **Agar Media** | **Color of colony** | **Margin of Colony** | **Colony Surface** | **Consistency** | **Opacity** |
| BEMB | EMB | Black | Entire | Convex | Moist | Iridescent |
| BEMCPC | EMB | Colorless Pink Centered | Entire | Convex | Mucoid | Opaque |
| BEMMS1 | EMB | Metallic Sheen | Entire | Convex | Moist | Iridescent |
| BEMMS2 | EMB | Metallic Sheen | Entire | Convex | Moist | Iridescent |
| BEMPM | EMB | Pinkish | Entire | Convex | Mucoid | Opaque |
| BMCLPIS | MacConkey | Light Purple | Irregular | Flat | Moist | Transparent |
| BMCPG1 | MacConkey | Purple | Entire | Convex | Mucoid | Opaque |
| BMCPG2 | MacConkey | Purple | Entire | Convex | Mucoid | Opaque |
| BMCPIS | MacConkey | Purple | Irregular | Flat | Moist | Transparent |
| BMCPM | MacConkey | Purple | Entire | Convex | Moist | Iridescent |
| BMCPWc | MacConkey | Purple White Centered | Irregular | Flat | Moist | Transparent |
| BMCT | MacConkey | Colorless | Irregular | Flat | Moist | Transparent |
| BMCTIS | MacConkey | Colorless | Irregular | Flat | Butyrous | Transparent |
| BMCWcP | MacConkey | Pink White Centered | Entire | Convex | Butyrous | Opaque |
| BMCWE | MacConkey | White | Entire | Convex | Butyrous | Opaque |
| BSSPuG | SS | Purple | Entire | Convex | Gummy | Iridescent |
| BSSPiG1 | SS | Pink Glossy | Entire | Convex | Moist | Opaque |
| BSSPiG2 | SS | Pink Glossy | Entire | Convex | Moist | Opaque |
| BSSPLF | SS | Light Purple | Entire | Flat | Moist | Transparent |
| BSSPMe | SS | Pink | Entire | Convex | Moist | Opaque |
| BSSPMa | SS | Pink Matte | Entire | Convex | Moist | Opaque |
| BSSPS | SS | Pink | Entire | Convex | Moist | Translucent |
| BSSWS | SS | White | Entire | Convex | Moist | Opaque |
| BTCDY1 | TCBS | Diffusive Yellow | Entire | Convex | Moist | Translucent |
| BTCDY2 | TCBS | Diffusive Yellow | Entire | Convex | Moist | Translucent |
| BPCMO | PCA | White | Entire | Convex | Butyrous | Opaque |
| BPCOWVS | PCA | Off White | Entire | Convex | Mucoid | Opaque |
| BPCSIS | PCA | White | Irregular | Flat | Butyrous | Opaque |
| BPCWL | PCA | White | Entire | Convex | Butyrous | Opaque |
| BPCWVS1 | PCA | White | Entire | Convex | Butyrous | Opaque |
| BPCWVS2 | PCA | White | Entire | Convex | Butyrous | Opaque |
| BPCYIS | PCA | Yellow | Irregular | Flat | Butyrous | Opaque |
| BPCWMe | PCA | White | Entire | Convex | Butyrous | Opaque |
| BPCWMa | PCA | White Matte | Entire | Convex | Butyrous | Opaque |
| BPCWG | PCA | White Glossy | Entire | Convex | Butyrous | Translucent |
| BPCWVS | PCA | White | Entire | Convex | Butyrous | Opaque |

| Supplementary Table 3: Colony characteristics of the isolates from Chicken Curry | | | | | | |
| --- | --- | --- | --- | --- | --- | --- |
| **Isolates** | **Agar Media** | **Color of colony** | **Margin of Colony** | **Colony Surface** | **Consistency** | **Opacity** |
| CEMPuG | EMB | Pink | Entire | Convex | Gummy | Iridescent |
| CEMPiG | EMB | Purple | Entire | Convex | Gummy | Iridescent |
| CEMPuM | EMB | Purple | Entire | Convex | Butyrous | Opaque |
| CEMPiM | EMB | Pink | Entire | Convex | Butyrous | Translucent |
| CEMC | EMB | Colorless | Irregular | Flat | Butyrous | Transparent |
| CMCPE | MacConkey | Pink | Entire | Convex | Butyrous | Translucent |
| CMCPiG | MacConkey | Pink Glossy | Entire | Convex | Butyrous | Translucent |
| CMCPuG | MacConkey | Purple | Entire | Convex | Gummy | Opaque |
| CMCPIS | MacConkey | Purple | Irregular | Flat | Moist | Transparent |
| CMCPL | MacConkey | Pink | Entire | Convex | Butyrous | Opaque |
| CMCWM | MacConkey | White | Entire | Convex | Butyrous | Opaque |
| CPCWM1 | PCA | White | Entire | Convex | Butyrous | Opaque |
| CPCWM2 | PCA | White | Entire | Convex | Butyrous | Opaque |
| CPCWS | PCA | White | Entire | Convex | Butyrous | Opaque |
| CSSPuL | SS | Purple Light | Entire | Convex | Gummy | Translucent |
| CSSPiL | SS | Pink | Entire | Convex | Gummy | Translucent |
| CSSPM | SS | Purple Matte | Entire | Convex | Gummy | Iridescent |
| CSSWS | SS | White | Entire | Convex | Butyrous | Opaque |
| CPCOWM | PCA | Off White | Entire | Convex | Mucoid | Opaque |
| CPCWLG | PCA | White | Entire | Convex | Butyrous | Opaque |
| CPCWO | PCA | White | Entire | Convex | Butyrous | Opaque |
| CPCWS1 | PCA | White | Entire | Convex | Butyrous | Opaque |
| CPCWS2 | PCA | White | Entire | Convex | Butyrous | Opaque |
| CPCYIS | PCA | Yellow | Irregular | Flat | Moist | Translucent |
| CPCMW | PCA | Milky White | Entire | Convex | Butyrous | Opaque |
| CPCWM | PCA | White Matte | Entire | Convex | Mucoid | Opaque |

| Supplementary Table 4: Morphological Characteristics of the Isolates from Potato  Smash | | | |
| --- | --- | --- | --- |
| **Code of the isolates** | **Morphology** | **Gram Reaction** | **Arrangement** |
| BEMB | Rod | Negative | Single-cell |
| BEMMS1 | Rod | Negative | Single-cell |
| BMCPG | Rod | Negative | Single-cell |
| BMCPIS | Rod | Negative | Clustered |
| BMCPM | Rod | Negative | Single-cell |
| BMCT | Rod | Negative | Single-cell |
| BMCTIS | Rod | Negative | Single-cell |
| BMCWcP | Rod | Negative | Single-cell |
| BSSPG1 | Rod | Negative | Single-cell |
| BSSPG2 | Rod | Negative | Single-cell |
| BSSPM | Rod | Negative | Single-cell |
| BSSPS | Rod | Negative | Clustered |
| BTCDY1 | Rod | Negative | Single-cell |
| BTCDY2 | Rod | Negative | Single-cell |
| BEMCPC | Rod | Negative | Single-cell |
| BEMMS2 | Rod | Negative | Single-cell |
| BEMPM | Rod | Negative | Single-cell |
| BMCLPIS | Rod | Negative | Single-cell |
| BMCPG | Rod | Negative | Single-cell |
| BMCPWc | Rod | Negative | Single-cell |
| BMCWE | Rod | Negative | Single-cell |
| BPCMO | Long Rod | Positive | Single-cell |
| BPCOWVS | Coccus | Positive | Single-cell |
| BPCSIS | Coccus | Positive | Single-cell |
| BPCWG | Coccus | Positive | Single-cell |
| BPCWL | Coccus | Positive | Clustered |
| BPCWMe | Coccus | Positive | Single-cell |
| BPCWMa | Long Rod | Positive | Single-cell |
| BPCWVS1 | Coccus | Positive | Single-cell |
| BPCWVS2 | Coccus | Positive | Single-cell |
| BPCWVS | Coccus | Positive | Single-cell |
| BPCYIS | Coccus | Positive | Single, Diplo, Chain |
| BSSPiG | Rod | Negative | Clustered |
| BSSPLF | Rod | Negative | Single-cell |
| BSSPM | Rod | Negative | Single-cell |
| BSSWS | Rod | Negative | Single-cell |

| Supplementary Table 5: Morphological Characteristics of the Isolates from Chicken Curry | | | |
| --- | --- | --- | --- |
| **Code of the isolates** | **Morphology** | **Gram Reaction** | **Arrangement** |
| CEMPG | Rod | Negative | Single-cell |
| CEMPMe | Rod | Negative | Single-cell |
| CMCPG | Rod | Negative | Single-cell |
| CMCWM | Rod | Negative | Single-cell |
| CPCWM | Rod | Negative | Single-cell |
| CSSPuL | Rod | Negative | Single-cell |
| CSSPM | Rod | Negative | Clustered |
| CPCMW | Coccus | Positive | Single-cell |
| CPCOWM | Coccus | Positive | Single-cell |
| CPCWLG | Long Rod | Positive | Single-cell |
| CPCWM | Long Rod | Positive | Single-cell |
| CPCWO | Long Rod | Positive | Single-cell |
| CPCWS1 | Coccus | Positive | Double cell |
| CPCWS2 | Coccus | Positive | Chain |
| CPCYIS | Coccus | Positive | Single-cell |
| CEMPuG | Rod | Negative | Single-cell |
| CEMPM | Rod | Negative | Single-cell |
| CEMT | Rod | Negative | Single-cell |
| CMCPE | Rod | Negative | Clustered |
| CMCPuG | Rod | Negative | Clustered |
| CMCPIS | Rod | Negative | Single-cell |
| CMCPL | Rod | Negative | Single-cell |
| CPCWM | Rod | Negative | Single-cell |
| CPCWS | Rod | Negative | Single-cell |
| CSSPiL | Rod | Negative | Single-cell |
| CSSWS | Rod | Negative | Single-cell |

| Supplementary Table 6: Biochemical Characteristics of the Gram-Negative Bacteria Isolated from Potato Smash and Chicken Curry Samples | | | | | | | | | | | |
| --- | --- | --- | --- | --- | --- | --- | --- | --- | --- | --- | --- |
| **Code Name** | **Gram Staining** | **KIA** | | | | **MIU** | | | **Oxidase** | **Catalase** | **Presumed Organism** |
|  |  | Slant | Butt | H2S | Gas | M | I | U |  |  |  |
| CEMPuG | (-) Rod | (-) | (+) | (-) | (+) | (+) | (+) | (-) | (-) | (+) | *E. coli* |
| CMCPIS | (-) Rod | (+) | (+) | (-) | (+) | (+) | (-) | (-) | (-) | (+) | *Enterobacter* |
| CMCPL | (-) Rod | (-) | (+) | (-) | (+) | (+) | (+) | (+) | (-) | (+) | *Proteus* |
| CMCPE | (-) Rod Clustered | (+) | (+) | (-) | (+) | (+) | (+) | (+) | (-) | (+) | *Klebsiella* |
| CMCPG | (-) Rod Clustered | (+) | (+) | (-) | (+) | (+) | (+) | (+) | (-) | (+) | *Klebsiella* |
| CSSPuL | (-) Rod | (-) | (+) | (-) | (+) | (+) | (-) | (-) | (-) | (+) | *Salmonella* |
| CSSWS | (-) Rod | (-) | (+) | (-) | (+) | (+) | (-) | (-) | (-) | (+) | *Salmonella* |
| CPCWM1 | (-) Rod | (-) | (+) | (-) | (+) | (+) | (+) | (-) | (-) | (+) | *E. coli* |
| CPCWS | (-) Rod | (-) | (+) | (-) | (+) | (+) | (+) | (-) | (-) | (+) | *E. coli* |
| BMCPWc | (-) Rod | (+) | (+) | (-) | (+) | (+) | (-) | (-) | (-) | (+) | *Enterobacter* |
| BMCWE | (-) Rod | (+) | (+) | (-) | (+) | (+) | (+) | (+) | (-) | (+) | *Klebsiella* |
| BMCLPIS | (-) Rod | (-) | (+) | (-) | (+) | (+) | (+) | (+) | (-) | (+) | *Proteus* |
| BMCPG1 | (-) Rod | (-) | (+) | (-) | (+) | (+) | (+) | (+) | (-) | (+) | *E. coli* |
| BSSWS | (-) Rod | (-) | (+) | (-) | (+) | (+) | (-) | (-) | (-) | (+) | *Salmonella* |
| BSSPMe | (-) Rod | (-) | (+) | (-) | (+) | (+) | (+) | (+) | (-) | (+) | *E. coli* |
| BSSPLF | (-) Rod | (-) | (+) | (-) | (+) | (+) | (+) | (+) | (-) | (+) | *Proteus* |
| BEMMS1 | (-) Rod | (+) | (+) | (-) | (+) | (+) | (+) | (-) | (-) | (+) | *E. coli* |
| BEMCPC | (-) Rod | (-) | (+) | (-) | (+) | (+) | (+) | (+) | (-) | (+) | *E. coli* |
| BEMPM | (-) Rod | (-) | (+) | (-) | (+) | (+) | (+) | (-) | (-) | (+) | *E. coli* |
| CEMPiM | (-) Rod | (-) | (+) | (-) | (+) | (+) | (+) | (-) | (-) | (+) | *E. coli* |
| CEMT | (-) Rod | (-) | (+) | (-) | (+) | (+) | (+) | (+) | (-) | (+) | *Proteus* |
| BSSPiG | (-) Rod Clustered | (-) | (+) | (-) | (+) | (+) | (-) | (-) | (-) | (+) | *Salmonella* |
| BSSPS | (-) Rod Clustered | (-) | (+) | (-) | (+) | (+) | (-) | (-) | (-) | (+) | *Salmonella* |
| BEMB | (-) Rod | (-) | (+) | (-) | (+) | (+) | (+) | (-) | (-) | (+) | *E. coli* |
| BMCPIS | (-) Rod Clustered | (+) | (+) | (-) | (+) | (+) | (-) | (-) | (-) | (+) | *Salmonella* |
| BMCTIS | (-) Rod | (-) | (+) | (-) | (+) | (+) | (+) | (+) | (-) | (+) | *Proteus* |
| CMCPG | (-) Rod | (-) | (+) | (-) | (+) | (+/-) | (+) | (+) | (-) | (+) | *Klebsiella* |
| CMCWM | (-) Rod | (-) | (+) | (-) | (+) | (+) | (+) | (-) | (+) | (+) | *Vibrio* |
| CSSPiL | (-) Rod | (+) | (+) | (-) | (+) | (+) | (-) | (-) | (-) | (+) | *Enterobacter* |
| CSSPM | (-) Rod Clustered | (-) | (+) | (-) | (+) | (+) | (+) | (+) | (-) | (+) | *Proteus* |
| CPCWM | (-) Rod | (-) | (+) | (-) | (+) | (-) | (+) | (+) | (-) | (+) | *Klebsiella* |
| CEMPiM | (-) Rod | (+) | (+) | (-) | (+) | (+) | (+) | (-) | (-) | (+) | *E. coli* |
| CEMPiG | (-) Rod | (+) | (+) | (-) | (+) | (-) | (+) | (+) | (-) | (+) | *Klebsiella* |
| BSSPuG | (-) Rod | (+) | (+) | (-) | (+) | (+) | (-) | (-) | (-) | (+) | *Enterobacter* |
| BSSPMa | (-) Rod | (+) | (+) | (-) | (+) | (+) | (+) | (-) | (-) | (+) | *E. coli* |
| BMCWcP | (-) Rod | (-) | (+) | (-) | (+) | (+) | (-) | (-) | (-) | (+) | *Enterobacter* |
| BMCT | (-) Rod | (+) | (+) | (-) | (+) | (+) | (+) | (+) | (-) | (+) | *Proteus* |
| BMCPG2 | (-) Rod | (-) | (+) | (-) | (+) | (+) | (+) | (-) | (-) | (+) | *E. coli* |
| BEMMS2 | (-) Rod | (-) | (+) | (-) | (+) | (+) | (+) | (-) | (-) | (+) | *E. coli* |
| BMCPM | (-) Rod | (+) | (+) | (-) | (+) | (+) | (-) | (+) | (-) | (+) | *Citrobacter* |
| BTCDY1 | (-) Rod | (-) | (=) | (-) | (+) | (+) | (+) | (+) | (+) | (+) | *Vibrio* |
| BTCDY2 | (-) Rod | (-) | (+) | (-) | (+) | (+) | (+) | (+) | (+) | (+) | *Vibrio* |
| BSSPiG2 | (-) Rod | (-) | (+) | (-) | (+) | (+) | (+) | (-) | (-) | (+) | *Salmonella* |

| Supplementary Table 7: Biochemical Characteristics of the Gram-Positive Bacteria Isolated from Potato Smash and Chicken Curry Samples | | | | | | | | | |
| --- | --- | --- | --- | --- | --- | --- | --- | --- | --- |
| **Code Name** | **Gram Staining** | **MR** | **VP** | **MIU** | | | **Oxidase** | **Catalase** | **Presumed Organism** |
|  |  |  |  | **M** | **I** | **U** |  |  |  |
| CPCWS1 | (+) Coccus (Diplo) | (=) | (-) | (-) | (-) | (+) | (-) | (+) | *Staphylococcus* |
| BPCWVS1 | (+) Coccus | (+) | +/- | (+) | (+) | (+) | (+) | (-) | *Streptococcus* |
| CPCOWM | (+) Coccus | (+) | (-) | (-) | (-) | (+) | (+) | (+) | *Micrococcus* |
| CPCWLG | (+) Long Rod | (-) | (-) | (+) | (-) | (-) | (-) | (-) | *Clostridium* |
| BPCOWVS | (+) Coccus | (-) | (-) | (-) | (-) | (+) | (-) | (+) | *Staphylococcus* |
| BPCWL | (+) Coccus (Clustered) | (+) | (-) | (-) | (-) | (+) | (+) | (+) | *Micrococcus* |
| BPCWVS2 | (+) Coccus | (+) | (-) | (+) | (+) | (+) | (+) | (-) | *Streptococcus* |
| BPCMO | (+) Long Rod | (+) | (+) | (+) | (-) | (+) | (+) | (-) | *Bacillus* |
| BPCSIS | (+) Coccus | (+) | (-) | (-) | (-) | (+) | (+) | (+) | *Micrococcus* |
| CPCWO | (+) Long Rod | (+) | (+) | (+) | (-) | (+) | (+) | (-) | *Bacillus* |
| CPCWS2 | (+) Coccus (Chain) | (+) | (-) | (+) | (+) | (+) | (+) | (-) | *Streptococcus* |
| CPCYIS | (+) Coccus | (+) | (-) | (+/-) | (+/-) | (+) | (-) | (+) | *Sarcina* |
| BPCYIS | (+) Coccus (Single, Diplo, chain) | (+) | (-) | (+/-) | (+) | (+) | (-) | (+) | *Sarcina* |
| BPCWVS | (+) Coccus | (+) | (-) | (+) | (+) | (+) | (+) | (-) | *Streptococcus* |
| CPCMW | (+) Coccus | (-) | (-) | (-) | (-) | (+/-) | (-) | (+) | *Planococcus* |
| CPCWM2 | (+) Long Rod | (+) | (+) | (+) | (-) | (+) | (+) | (-) | *Bacillus* |
| BPCWM | (+) Long Rod | (+) | (+) | (+) | (-) | (+) | (+) | (-) | *Bacillus* |
| BPCWG | (+) Coccus | (-) | (-) | (-) | (-) | (+) | (-) | (+) | *Staphylococcus* |
| BPCWMa | (+) Coccus | (-) | (-) | (-) | (-) | (-) | (-) | (+) | *Staphylococcus* |

| Supplementary Table 8: Identified Organisms in Potato Smash Samples from Different Food Delivery Points | | | |
| --- | --- | --- | --- |
| **Code of the canteen** | **Code Name** | **Morphology** | **Presumed Organism** |
| Canteen A | BEMCPC | Gram Negative | *E. coli* |
| Canteen A | BEMMS1 | Gram Negative | *E. coli* |
| Canteen A | BMCLPIS | Gram Negative | *Proteus* |
| Canteen A | BMCPG1 | Gram Negative | *E. coli* |
| Canteen A | BMCPWc | Gram Negative | *Enterobacter* |
| Canteen A | BMCWE | Gram Negative | *Klebsiella* |
| Canteen A | BSSPLF | Gram Negative | *Proteus* |
| Canteen A | BSSPMe | Gram Negative | *E. coli* |
| Canteen A | BSSWS1 | Gram Negative | *Salmonella* |
| Canteen A | BPCWVS1 | Gram Positive | *Streptococcus* |
| Canteen B | BEMPM | Gram Negative | *E. coli* |
| Canteen B | BPCOWVS | Gram Positive | *Staphylococcus* |
| Canteen B | BPCWL | Gram Positive | *Micrococcus* |
| Canteen C | BSSPiG | Gram Negative | *Salmonella* |
| Canteen C | BSSPS | Gram Negative | *Salmonella* |
| Canteen C | BEMB | Gram Negative | *E. coli* |
| Canteen C | BMCPIS | Gram Negative | *Salmonella* |
| Canteen C | BMCTIS | Gram Negative | *Proteus* |
| Canteen C | BPCWVS2 | Gram Positive | *Streptococcus* |
| Canteen C | BPCMO | Gram Positive | *Bacillus* |
| Canteen C | BPCSIS | Gram Positive | *Micrococcus* |
| Canteen D | BEMMS2 | Gram Negative | *E. coli* |
| Canteen D | BMCPG2 | Gram Negative | *E. coli* |
| Canteen D | BMCT | Gram Negative | *Proteus* |
| Canteen D | BMCWcP | Gram Negative | *Enterobacter* |
| Canteen D | BSSPMa | Gram Negative | *E. coli* |
| Canteen D | BSSPG1 | Gram Negative | *E. coli* |
| Canteen D | BPCYIS | Gram Positive | *Sarcina* |
| Canteen D | BPCWMe | Gram Positive | *Staphylococcus* |
| Canteen D | BPCWVS | Gram Positive | *Streptococcus* |
| Canteen E | BMCPM | Gram Negative | *Citrobacter* |
| Canteen E | BTCDY1 | Gram Negative | *Vibrio* |
| Canteen E | BTCDY2 | Gram Negative | *Vibrio* |
| Canteen E | BSSPG2 | Gram Negative | *Salmonella* |
| Canteen E | BPCWMa | Gram Positive | *Bacillus* |
| Canteen E | BPCWG | Gram Positive | *Staphylococcus* |

| Supplementary Table 9: Identified Organisms in Chicken Curry Samples from Different Food Delivery Points | | | |
| --- | --- | --- | --- |
| **Code of the canteen** | **Code Name** | **Morphology** | **Presumed Organism** |
| Canteen A | CEMPuG | Gram Negative | *E. coli* |
| Canteen A | CMCPE | Gram Negative | *Klebsiella* |
| Canteen A | CMCPG | Gram Negative | *Klebsiella* |
| Canteen A | CMCPIS | Gram Negative | *Enterobacter* |
| Canteen A | CMCPL | Gram Negative | *Proteus* |
| Canteen A | CPCWM | Gram Negative | *E. coli* |
| Canteen A | CPCWS | Gram Negative | *E. coli* |
| Canteen A | CSSPuL | Gram Negative | *Salmonella* |
| Canteen A | CSSWS | Gram Negative | *Salmonella* |
| Canteen A | CPCWS1 | Gram Positive | *Staphylococcus* |
| Canteen B | CEMPuM | Gram Negative | *E. coli* |
| Canteen B | CEMT | Gram Negative | *Proteus* |
| Canteen B | CPCOWM | Gram Positive | *Micrococcus* |
| Canteen B | CPCWLG | Gram Positive | *Clostridium* |
| Canteen C | CPCWO | Gram Positive | *Bacillus* |
| Canteen D | CSSPiL | Gram Negative | *Enterobacter* |
| Canteen D | CSSPM | Gram Negative | *Proteus* |
| Canteen D | CMCPiG | Gram Negative | *E. coli* |
| Canteen D | CMCWM | Gram Negative | *Vibrio* |
| Canteen D | CPCWM | Gram Negative | *Klebsiella* |
| Canteen D | CEMPiG | Gram Negative | *Klebsiella* |
| Canteen D | CEMPiM | Gram Negative | *E. coli* |
| Canteen D | CPCWS2 | Gram Positive | *Streptococcus* |
| Canteen D | CPCYIS | Gram Positive | *Sarcina* |
| Canteen E | CPCMW | Gram Positive | *Planococcus* |
| Canteen E | CPCWM | Gram Positive | *Bacillus* |

| Supplementary Table 10: Antibiotic Resistance Analysis by Disk Diffusion Method of Gram-Negative Isolates (26) Following Interpretive Standard | | | | | | | | | | | | | | | | |  |
| --- | --- | --- | --- | --- | --- | --- | --- | --- | --- | --- | --- | --- | --- | --- | --- | --- | --- |
| **Presumed Organism** | **AZM** | | **CIP** | | **TE** | | **AMP** | | **LE** | | **C** | | **CL** | | **GEN** | |  |
| *E. coli* | INT | | S | | R | | R | | S | | S | | INT | | S | |  |
| *Enterobacter* | R | | S | | R | | R | | S | | S | | R | | S | |  |
| *Proteus* | S | | S | | INT | | R | | S | | S | | INT | | S | |  |
| *Klebsiella* | INT | | S | | S | | R | | S | | S | | INT | | S | |  |
| *Salmonella* | S | | S | | INT | | R | | S | | S | | INT | | S | |  |
| *E. coli* | S | | S | | R | | R | | S | | S | | INT | | S | |  |
| *Klebsiella* | INT | | S | | S | | R | | S | | S | | R | | S | |  |
| *Proteus* | INT | | S | | INT | | R | | S | | S | | R | | S | |  |
| *Enterobacter* | R | | S | | INT | | R | | S | | S | | R | | S | |  |
| *E. coli* | INT | | S | | S | | R | | S | | S | | INT | | S | |  |
| *E. coli* | R | | S | | R | | R | | S | | S | | INT | | S | |  |
| *E. coli* | S | | S | | INT | | R | | S | | S | | INT | | S | |  |
| *Salmonella* | R | | S | | INT | | R | | S | | S | | R | | S | |  |
| *E. coli* | S | | S | | S | | R | | S | | S | | INT | | S | |  |
| *Salmonella* | S | | S | | S | | R | | S | | S | | R | | S | |  |
| *Klebsiella* | R | | S | | S | | R | | S | | S | | R | | S | |  |
| *Vibrio* | S | | S | | S | | R | | S | | S | | S | | S | |  |
| *Proteus* | S | | R | | R | | R | | R | | S | | R | | S | |  |
| *E. coli* | INT | | S | | S | | R | | S | | S | | R | | S | |  |
| *Proteus* | INT | | S | | INT | | R | | S | | S | | INT | | S | |  |
| *E. coli* | INT | | S | | INT | | R | | S | | S | | R | | S | |  |
| *E. coli* | INT | | S | | S | | R | | S | | S | | INT | | S | |  |
| *Citrobacter* | R | | S | | R | | R | | S | | S | | R | | S | |  |
| *Vibrio* | INT | | S | | S | | R | | S | | S | | S | | S | |  |
| *Vibrio* | R | | S | | INT | | R | | S | | S | | INT | | S | |  |
| *Salmonella* | R | | S | | S | | R | | S | | S | | INT | | S | |  |
| *R= Resistant, INT= Intermediate, S= Sensitive | | | | | | | | | | | | | | | | |  |
| AZM=Azithromycin 30 µg, CIP= Ciprofloxacin 5 µg, TE=Tetracycline 30 µg, AMP=Ampicillin 25 µg, LE=Levofloxacin 5 µg, C= Chloramphenicol 30 µg, CL=Colistin 10 µg, GEN=Gentamycin 10 µg | | | | | | | | | | | | | | | | |  |
| **Control Organisms** | **AZM** | | **CIP** | | **TE** | | **AMP** | | **LE** | | **C** | | **CL** | | **GEN** | |  |
| *E. coli* | S | | S | | S | | R | | S | | S | | R | | S | |  |
| *Shigella* | S | | INT | | R | | R | | INT | | S | | S | | S | |  |
| *Salmonella* | S | | R | | R | | R | | R | | S | | S | | INT | |  |
| *Klebsiella* | INT | | S | | S | | R | | S | | S | | INT | | S | |  |
| *Pseudomonas* | S | | S | | S | | R | | S | | S | | R | | INT | |  |
| *Proteus* | R | | INT | | R | | R | | S | | R | | R | | S | |  |
| *Vibrio* | S | | S | | S | | INT | | S | | S | | R | | S | |  |
| *Enterobacter* | S | | S | | S | | R | | S | | S | | R | | S | |  |
| **R= Resistant, INT= Intermediate, S= Sensitive* | | | | | | | | | | | | | | | | |  |
| Supplementary Table 11: Antibiotic Resistance Analysis by Disk Diffusion Method of Gram-Positive Isolates (18) Following Interpretive Standard | | | | | | | | | | | | | | | | |  |
| **Presumed Organisms** | | **AZM** | | **CIP** | | **TE** | | **AMP** | | **LE** | | **C** | | **CL** | | **GEN** | |
| *Staphylococcus* | | S | | S | | S | | R | | S | | S | | INT | | S | |
| *Bacillus* | | S | | S | | S | | R | | S | | S | | INT | | S | |
| *Micrococcus* | | S | | S | | S | | R | | S | | S | | INT | | S | |
| *Clostridium* | | S | | S | | R | | R | | S | | S | | R | | S | |
| *Staphylococcus* | | S | | S | | S | | R | | S | | INT | | S | | S | |
| *Micrococcus* | | S | | S | | S | | R | | S | | S | | R | | S | |
| *Streptococcus* | | S | | S | | S | | R | | S | | S | | R | | S | |
| *Bacillus* | | S | | S | | S | | R | | S | | S | | R | | S | |
| *Micrococcus* | | R | | S | | R | | R | | S | | S | | INT | | S | |
| *Streptococcus* | | R | | S | | R | | R | | S | | S | | R | | S | |
| *Sarcina* | | R | | S | | R | | INT | | S | | S | | R | | S | |
| *Sarcina* | | S | | S | | S | | S | | S | | S | | INT | | S | |
| *Staphylococcus* | | INT | | S | | S | | R | | S | | INT | | INT | | INT | |
| *Streptococcus* | | S | | S | | S | | INT | | S | | INT | | R | | S | |
| *Planococcus* | | R | | S | | S | | R | | S | | S | | R | | S | |
| *Bacillus* | | S | | S | | S | | R | | S | | S | | R | | S | |
| *Bacillus* | | S | | S | | S | | R | | S | | S | | R | | S | |
| *Staphylococcus* | | S | | S | | S | | R | | S | | R | | R | | S | |
| **R= Resistant, INT= Intermediate, S= Sensitive* | | | | | | | | | | | | | | | | | |
| AZM=Azithromycin 30 µg, CIP= Ciprofloxacin 5 µg, TE=Tetracycline 30 µg, AMP=Ampicillin 25 µg, LE=Levofloxacin 5 µg, C= Chloramphenicol 30 µg, CL=Colistin 10 µg, GEN=Gentamycin 10 µg | | | | | | | | | | | | | | | | | |
|  | |  | |  | |  | |  | |  | |  | |  | |  | |
| **Control Organisms** | | **AZM** | | **CIP** | | **TE** | | **AMP** | | **LE** | | **C** | | **CL** | | **GEN** | |
| *Bacillus* | | S | | S | | S | | R | | S | | S | | R | | S | |
| *Streptococcus* | | S | | S | | S | | R | | S | | S | | INT | | S | |
| *Staphylococcus* | | S | | S | | S | | R | | S | | S | | R | | S | |
| *R= Resistant, INT= Intermediate, S= Sensitive | | | | | | | | | | | | | | | | | |

| Supplementary Table 12: Composition of Master Mix for PCR | | | |
| --- | --- | --- | --- |
| EPEC & ETEC Reaction Mixture | | EIEC & EHEC Reaction Mixture | |
| Reagents | Volume in microliter | Reagents | Volume in microliter |
| Autoclaved distilled water | 7.5 | Autoclaved distilled water | 7.5 |
| Magnesium chloride | 0.25 | Magnesium chloride | 0.25 |
| Dimethyl sulfoxide (DMSO) | 0.25 | Dimethyl sulfoxide (DMSO) | 0.25 |
| Forward Primer 1 | 0.75 | Forward Primer | 1.25 |
| Reverse Primer 1 | 0.75 | Reverse Primer | 1.25 |
| Forward Primer 2 | 0.75 | Master mix | 12.5 |
| Reverse Primer 2 | 0.75 | Template DNA | 2 |
| Master mix | 12.5 | Total Volume | 25 |
| Template DNA | 1.5 | *DMSO was used to inhibit non-specific binding to enhance specificity. Magnesium chloride salt worked as a co-factor of the tec DNA polymerase enzyme to enhance efficiency. | |
| Total Volume | 25 |  |  |
